# The predictive effects of foreign language anxiety and boredom on willingness to communicate among Chinese struggling EFL learners

**DOI:** 10.1016/j.heliyon.2023.e19610

**Published:** 2023-08-29

**Authors:** Shuxia Bai

**Affiliations:** School of College English Teaching and Research, Henan University, Kaifeng, Henan Province, PR China

**Keywords:** Willingness to communicate, Foreign language anxiety, Foreign language boredom, Foreign language learning, Chinese struggling EFL learners

## Abstract

Language learners' willingness to communicate (WTC) plays a critical role in learning English as a foreign language (EFL), and affects the learning outcome. However, the WTC of struggling EFL learners remains largely uninvestigated. Thus, the present study aimed to scrutinize the correlation between foreign language anxiety (FLA), foreign language boredom (FLB) and WTC, and the predictor role of FLA and FLB in WTC in the context of Chinese struggling EFL learners. A total of 662 participants majoring in music, physical education and fine arts from four universities in China attended this study. Pearson correlation and a Structural Equation Modeling (SEM) were used to substantiate that these struggling students' FLA was highly and positively correlated with their FLB, while both their FLA and FLB were negatively linked to WTC and significantly predicted their WTC. In discussion and conclusion, the consistency between the current study and related studies, some limitations as well as further implications are presented for avid researchers in future EFL study of those who have difficulty in learning English.

## 1 Introduction

Emotions affect students' learning and performance and permeate school education. However, for a long time, there has been a neglect of emotions in the realm of second language acquisition (SLA), which has conventionally deemed emotions irrational factors [1]. With the introduction and prevalence of positive psychology (PP) movement in the past decade, nevertheless, there is a rising interest in emotion research in foreign and second language learning and teaching settings [2,3]. A broad range of emotions, both positive and negative, have been attended to aside from the most extensively studied anxiety [4,5]. Compared with foreign scholars, Chinese scholars started the study of emotions much later.

Anxiety is a prevalent feeling existing in the process of foreign language learning. Since the 1970s, anxiety has become one of the hot topics in the study of second language acquisition abroad, but in China it only received attention in the early 21st century. Horwitz [6] states that most learners are subjected to foreign language anxiety (FLA) in various levels. Moreover, it is widely believed that anxiety generated in the learning process is one of the main obstacles learners confront when learning a foreign language [7,8]. Krashen [9] believes that FLA prevents information from entering the language acquisition area of the brain. Arnold and Brown [10] find that FLA can suppress a learner's learning ability and render the best teaching methods and materials ineffective. The effects of FLA on foreign language learning are intricate and multidimensional [11], but it is generally agreed that FLA acts as a key predictor of success and failures in foreign language (FL) learning [12]. High level of FLA usually results in a poor academic achievement and performance, which has been manifested by Horwitz [6]. As a challenging issue in FL learning, FLA still needs more research and exploration [13].

As a kind of suppressed negative emotion, boredom seriously hinders the stimulation and maintenance of students' learning motivation, thus affecting the learning effect [14,15]. Its devastating effects on learning have provoked abundant studies in educational psychology [16]. However, scant attention has been paid to the study of boredom in the learning of a second/foreign language (L2/FL), especially compared with other well-researched emotions such as anxiety and enjoyment [15,17]. Research on boredom in foreign language learning started with Chapman's study of boredom in the German foreign language classroom in 2013 [18], while in China it was not until 2020 that some scholars began to conduct relevant studies on foreign language learners' boredom [5,15,[19], [20], [21]]. Foreign Language Boredom (FLB) is a complex dynamic system [5,22], which may have the characteristics of general learning boredom, such as lack of interest, distraction, sleepiness, weariness and tiredness, but it is more closely related to the unique learning environment of a foreign language [20]. At different phases of learning a foreign language, boredom may be affected by various factors both individual (motivation, anxiety, etc.) and environmental (learning content, learning tasks, teacher-student interaction, etc.) [18,22]. Current research so far on FLB mainly focuses on exploring the conceptual structure, measurement tools and influencing factors [5,22], and it is urgent to broaden the scope of research.

Language is for communication, thus language learners' willingness to communicate (WTC) is a research realm which has attracted a large number of researchers to study from a variety of dimensions. It has been identified that WTC plays a critical role in learning English as a second/foreign language (L2/EFL), and higher level of WTC is generally consistent with better learning outcomes [23,24]. However, with regard to learners’ emotions affecting their WTC, scholarly attention has mainly put on L2 anxiety and enjoyment [25,26]. The effects of other emotions, including boredom, on L2 WTC are still in need of exploration. Besides, the objects of WTC study are mostly normal undergraduate English and non-English majors [[27], [28], [29]], and postgraduate students [30], which need to extend to other EFL learners at different levels of language learning, especially the struggling EFL learners.

There is a special group of EFL learners in China － college students majoring in music, physical education, and fine arts. Those students are generally regarded as struggling EFL learners and they are faced with unique challenges compared to other EFL learners at colleges. The emergence of this phenomenon is related to the localized culture in China. The college entrance examination for those students prioritizes the scores obtained in their specialized majors, resulting in lower admission requirements for English and other non-major courses. Consequently, those students dedicate a significant amount of time and effort to the learning of their majors, often neglecting the study of English. As a result, after entering colleges, students in these majors generally exhibit a lower level of English proficiency, characterized by their limited vocabulary and insufficient capability in listening, speaking, reading and writing. Naturally, some negative emotions including anxiety and boredom arise in their process of English learning.

In this context, the present study endeavors to take these Chinese struggling EFL learners as the objects of study and investigate the interplay of their foreign language anxiety, boredom and WTC, and the predictive effects of foreign language anxiety and boredom on their WTC, which will be of significant importance in shedding light on the corresponding learning and teaching strategies to those in urgent need of help in EFL learning. Therefore, the current study sought to answer the following research questions:

1. Is there any link between foreign language anxiety, foreign language boredom and willingness to communicate in Chinese struggling EFL learners?
2. How can the willingness to communicate of Chinese struggling EFL learners be anticipated by their foreign language anxiety and foreign language boredom?

## 2 Literature review

### 2.1 Foreign language anxiety

Among the negative emotions associated with the learning of a foreign language (FL), anxiety is one of the most extensively studied realms. Horwitz et al. [31] defined foreign language anxiety (FLA) to be “a distinct complex of self-perceptions, beliefs, feelings, and behaviors related to classroom language learning arising from the uniqueness of the language learning process” (p.128). Maclntyre [12] described FLA as “the worry and negative emotional reaction aroused when learning or using a second language” (p.27).

A large number of studies have been carried out to explore the potential sources of FLA, which found that FLA arousal is related to many factors, from error correction [32], peer competitiveness [33], incompatibility between teacher and student [34] to personality traits such as perfectionism [35] and neuroticism [36]. Learners' perception about their foreign language proficiency may also lead to the occurrence of different levels of anxiety [37]. Besides, teachers' attitudes and personalities are significant in provoking FLA [38]. In terms of the effects of FLA on the four skills of learning a foreign language, many studies have proved that FLA is negatively correlated with listening performance [39], oral tests [40], reading performance [41] and writing level [42]. In regard to the connection with other emotions, Dewaele and MacIntyre [43] found that FLA and foreign language enjoyment (FLE) coexist in FL learning, with a significant negative correlation but independent of each other.

There is also some research on Chinese EFL learners' anxiety. A quasi-experimental study conducted by Jin et al. [44] demonstrated that contracting Chinese-*as*-the-first-language university students' speaking in the foreign language (FL) classroom is a feasible approach to reducing their FL anxiety, and can foster some positive mindsets and behaviors in FL learning. Randomly selecting 145 PRC students in Singapore as the sample, Zhang [45] did an exploratory study of these ESL students' general language learning anxiety in a study-abroad context. Another study conducted by Li [46] focused on the relation between emotional intelligence and English achievement through the mediating effects of enjoyment, anxiety and burnout among Chinese Year-2 senior high school students.

Despite the large number of studies on FLA from various perspectives, researchers still need to expand the research angles and research objects to have a deeper understanding of FLA.

### 2.2 Foreign language boredom

Boredom, one of the most disturbing negative emotions a learner experiences in learning process, was defined as a negative state of psychology accompanied by low physical arousal and cognitive activation [17,19,47]. It has been proven that boredom hinders performance and higher levels of boredom will lead to lower achievement [48]. Despite the prolific research findings on boredom from psychology and educational psychology, more efforts should be devoted into the study of boredom in the context of foreign language learning which is still under-researched [49,50]. Among the studies conducted so far, Zawodniak et al. [51] found that all the participants in the experiment (30 Polish English-major students) complained about being bored in their English class. Pawlak et al. [52] discovered that students' lack of creativity and enthusiasm, together with their negative attitudes, would aggravate the occurrence of boredom. Taking Thai university students as the objects of study, Nakamura et al. [53] revealed some key elements leading to the arousal of boredom: learners' lamentable comprehensive ability, inadequate language proficiency, difficult tasks, and mismatching between learners and learning activities. Learning content may also bring about different levels of boredom. The greater the connection between learning content and real life, the lower the boredom level of students, which has been confirmed by Pawlak et al. [54]. In a recent survey, Derakhshan et al. [55] explored the causes of and solutions to boredom among English major Iranian students in COVID-19 prompted online English classes.

In China, Chengchen Li can be viewed as the leading figure in studying Chinese students' foreign language boredom. Her research mainly focuses on online English learning. Li and Dewaele [15] examined the predictive effects of trait emotional intelligence and online learning achievement perceptions on foreign language class boredom among Chinese tertiary students through an online questionnaire on the basis of control-value theory. Li and Han [21] investigated the relation and effects of foreign language anxiety and boredom along with enjoyment on the learning outcomes of online English classrooms. More importantly, Li et al. [19] revealed the prevalent emotional experience of boredom among Chinese university students in learning English through conducting questionnaire survey and interviews with non-English major students and some teachers, thus conceptualized Foreign Language Boredom (FLB) and Foreign Language Learning Boredom (FLLB) and developed the Foreign Language Learning Boredom Scale (FLLBS), which provides a high-level measurement tool with high reliability and validity for the follow-up research and is used in the present study.

### 2.3 Willingness to communicate

The concept of willingness to communicate (WTC) was first proposed in the study of the first language (L1) communication. It began to draw researchers’ attention in second language (L2) acquisition in the 1990s. MacIntyre et al. [56] defined L2 WTC as “readiness to enter into discourse at a particular time with a specific person or persons, using a L2” (p.547) and claimed that L2 WTC should be “the ultimate goal of the learning process” (p.547). They found that L2 WTC was more complicated and situation-dependent than L1 WTC, building a multi-layer pyramid model to illustrate different variables influencing WTC.

Up to now, an increasing number of studies have shown the relation of WTC with many individual, socio-psychological, and contextual factors. MacIntyre et al. [56] proposed five personality traits affecting WTC: extraversion, agreeableness, conscientiousness, emotional stability, and openness to new experiences. The more extroverted a learner is, the more willing he is to communicate [57]. Learners' familiarity with topics under discussion also has a direct correlation with their WTC during class time [58,59]. In addition, teachers play an important role in determining students' WTC. Effective teaching strategies employed by teachers like praise and positive feedback can build rapport with students, thus stimulating their WTC in learning English [60]. Based on his previous research, Kruk [61] discovered that boredom is positively associated with foreign language anxiety, but negatively connected with learners' WTC and motivation. Besides, as regard to other factors affecting WTC, cultural value should not be ignored, although it is mostly reflected in Asian learners. As noted by Wen and Clément [62] and Osterman [63], Asian cultural value such as implicit expression, introvert personality and less tolerance of ambiguity has a strong effect on Asian learners' WTC during class. Anyhow, scant attention has been attended to the study of the correlation between WTC and different emotions, and the study objects of WTC should be expanded.

The current empirical study is aimed to bridge several research gaps in the existing literature. First, the correlation among the three variables － foreign language anxiety, boredom and willingness to communicate, has hardly been researched so far. Second, there has been neglect to the study of the struggling EFL learners worldwide. As to Chinese struggling EFL learners, less attention has been paid to them. The related studies carried out in China often take the advanced college students of top universities or normal students in other universities or high schools as the research objects, the struggling students are largely overlooked. However, the situation of struggling students in learning English is quite different from that of other students and is well worth studying. It's a pity that to date no empirical study has been conducted to explore the WTC of these struggling students. Thus, to fill these gaps, the present study is set to investigate the interplay of foreign language anxiety, boredom and WTC, and the predictive effects of foreign language anxiety and boredom on WTC, taking Chinese struggling EFL learners as the objects of study. Conducting the research holds significant importance and relevance as it has the potential to provide valuable insights and guidance to researchers working on struggling EFL learners. Moreover, the findings of this study can contribute to the development of effective learning and teaching strategies designed to address the needs of struggling students and benefit both students and their instructors.

## 3 Methodology

### 3.1 Participants

In this study, students participating in the questionnaire were 662 undergraduates (649 valid cases) majoring in music, physical education and fine arts from four comprehensive universities in China. As revealed in Table 1, participants in music major accounted for 34.67%, physical education 42.83%, and fine arts 22.50%. Most of them were freshmen (50.39%) and sophomores (48.84%), who were required to take English as a compulsory course, while the percentages of juniors (0.62%) and seniors (0.15%) were small, who no longer took English classes but were still learning English for various purposes, such as passing College English Test Band 6 and the postgraduate entrance examination. In terms of gender, 278 were males and 371 females, whose average age was 19 years old. The questionnaire was based on random sampling.

**Table 1:** Demographic information of participants.

Demographic Information Category	*N*	*(%)*
**Major**		
Music	225	(34.67)
Physical education	278	(42.83)
Fine arts	146	(22.50)
**Level of education**		
Freshmen	327	(50.39)
Sophomores	317	(48.84)
Juniors	4	(0.62)
Seniors	1	(0.15)
**Gender**		
Male	278	(42.84)
Female	371	(57.16)
**Average age**	19	
Total	649	(100)

### 3.2 Instruments

The participants' foreign language anxiety (FLA) was evaluated by the 8-item *Foreign Language Classroom Anxiety Scale* (FLCAS) [43], extracted from the original FLCAS with 33 items [31]. Responses are given on a standard 5-point Likert scale (strongly disagree = 1, disagree = 2, undecided = 3, agree = 4, strongly agree = 5). In line with the original FLCAS, 6 items are phrased to indicate high anxiety (a sample: “Even if I am well prepared for FL class, I feel anxious about it.”) and 2 items are phrased to reflect low anxiety (i.e. “I don't worry about making mistakes in FL class.”/“I feel confident when I speak in FL class.”). Since the two low anxiety items are reverse-coded, high scores in this scale still show high anxiety [43].

*Foreign Language Learning Boredom Scale* (FLLBS) was developed and validated by Li et al. [19]. *Foreign Language Classroom Boredom Subscale* (FLCBS) is one of its subscales with a high reliability (Alpha = 0.947), which was employed in this study to evaluate the foreign language boredom (FLB) of Chinese EFL learners majoring in music, physical education and fine arts. FLCBS consists of 8 items rated on the standard 5-point Likert scale, ranging from 1 (strongly disagree) to 5 (strongly agree). A sample item is as follows: “I am only physically in the classroom, while my mind is wandering outside the English class."

Besides, *Willingness to Communicate inside the Classroom Scale* designed by MacIntyre et al. [64] was utilized to estimate the degree of a student's willingness to communicate (WTC) during class time. French used in the original scale was changed to English to adapt to the research object in the present study. Participants are required to indicate the degree of their willingness to communicate on the 5-point scale (almost never willing = 1, sometimes willing = 2, willing half of the time = 3, usually willing = 4, almost always willing = 5). A total of 27 items are grouped into four skill areas, with 8 items in speaking (Alpha = 0.81), 6 items in reading (Alpha = 0.83), 8 items in writing (Alpha = 0.88), and 5 items in comprehension (Alpha = 0.83) [64]]. The item examples are from (1) “Speaking in a group about your summer vacation.” to (27) “Understand an English movie.”

### 3.3 Data collection procedure

The present study collected quantitative data through a questionnaire, which comprises five sections: the consent form of the participants, the basic information about them including age, school, gender, level of education and major, and three measures: *Foreign Language Classroom Anxiety Scale* (FLCAS), *Foreign Language Classroom Boredom Subscale* (FLCBS), and *Willingness to Communicate inside the Classroom Scale*. There are 43 items in total and it lasted for a month. First, since the objects of this study were Chinese college students majoring in music, physical education and fine arts who are generally viewed as struggling students in learning English, the items in the questionnaire were translated into Chinese to help the participants profoundly conceptualize the questions and answer them. The translated Chinese version was double-checked by two experts in the field of linguistics to ensure the validity of the data collection. Then, the questionnaire was generated into the electronic version through Wenjuanxing (a professional online questionnaire survey platform in China). Before the distribution of the three measures, participants were required to complete a written consent form based on moral considerations [65]. In this way, they knew about the nature and purpose of the research and became aware that their participation was anonymous, the collected data would be kept confidential only for research goals, and they had the right to withdraw from the survey without any negative consequences. Since all the participants understood the nature and purpose of the research and completed a written consent form before they voluntarily participated in it, the ethical approval of the questionnaire was waived by Henan University Academic and Research Committee. It is also worth mentioning that the e-version of the questionnaire was carried out via WeChat (a Chinese instant messaging service application) with the help of warm-hearted colleagues and friends who are the English teachers of the participants. It took approximately 5–8 min to fill in the questionnaire. During their English classes, these teachers distributed the questionnaire via WeChat to their students and spared about 10 min for them to voluntarily participate in it. A total of 662 students took part in the survey with 649 cases valid.

## 4 Results

The descriptive statistics of the obtained results are reported in Table 2. As Table 2 displays, 649 students participated in this study. Besides, the mean score of these students' willingness to communicate is identified as 81.17, while their foreign language anxiety has a mean score of 25.47 and boredom 20.47.

**Table 2:** Descriptive statistics of the FLA, FLB, and WTC.

	*N*	Minimum	Maximum	Mean	*SD*
Foreign language anxiety	649	8	40	25.47	4.97
Foreign language boredom	649	8	40	20.47	8.20
Willingness to communicate	649	27	135	81.17	23.47

Table 3 reveals the results from Cronbach alpha analyses, which show all the indexes of Cronbach alpha in the employed questionnaire are quite acceptable including the sub-constructs.

**Table 3:** Results of Cronbach alpha indexes.

*Scale*	*Subscales*	*Cronbach alpha*
Foreign language anxiety	–	0.88
Foreign language boredom	–	0.97
Willingness to communicate	Overall	0.98
	Speaking	0.94
	Reading	0.95
	Writing	0.97
	Comprehension	0.92

### 4.1 The first RQ

RQ1 Is there any link between foreign language anxiety, foreign language boredom and willingness to communicate in Chinese struggling EFL learners?

To find the answer to the first research question, Pearson Correlation was utilized. Table 4 demonstrates the consequences of Pearson Correlation between foreign language anxiety, foreign language boredom and willingness to communicate of Chinese EFL learners majoring in music, physical education and fine arts.

**Table 4:** Results of Pearson Correlation between FLA, FLB, and WTC.

		Foreign language anxiety	Foreign language boredom	Willingness to communicate
Foreign language anxiety	Pearson Correlation P-value N	1.00		
Foreign language boredom	Pearson Correlation P-value N	0.52^a^ 5.12 × 10^−47^ 649	1.00	
Willingness to communicate	Pearson Correlation P-value N	−0.26^a^ 1.51 × 10^−11^ 649	−0.45^a^ 1.26 × 10^−33^ 649	1.00

a Correlation is significant at the 0.01 level (two-tailed).

As can be seen in Table 4, there is a positive high connection between foreign language anxiety and foreign language boredom (r = 0.52, n = 649, p = 5.12 × 10^−47^, α = 0.01). The overall WTC is negatively linked with both students' foreign language anxiety (r = −0.26, n = 649, p = 1.51 × 10^−11^, α = 0.01) and foreign language boredom (r = −0.45, n = 649, p = 1.26 × 10^−33^, α = 0.01).

Table 5 presents the consequences of Pearson Correlation between all sub-constructs WTC and foreign language anxiety.

**Table 5:** Results of Pearson Correlation between all sub-constructs WTC and FLA.

		Speaking	Reading	Writing	Comprehension
Foreign language anxiety	Pearson Correlation P-value N	−0.24^a^ 2.83 × 10^−10^ 649	−0.24^a^ 2.83 × 10^−10^ 649	−0.24^a^ 6.45 × 10^−10^ 649	−0.25^a^ 1.21 × 10^−10^ 649

a Correlation is significant at the 0.01 level (two-tailed).

As Table 5 indicates, there are negative relationships between all sub-constructs WTC and foreign language anxiety: speaking (r = −0.24，n = 649, p = 2.83 × 10^−10^, α = 0.01), reading (r = −0.24, n = 649, p = 2.83 × 10^−10^, α = 0.01), writing (r = −0.24, n = 649, p = 6.45 × 10^−10^, α = 0.01), and comprehension (r = −0.25, n = 649, p = 1.21 × 10^−10^, α = 0.01).

Table 6 reveals the results of Pearson Correlation between all sub-constructs WTC and foreign language boredom.

**Table 6:** Results of Pearson Correlation between all sub-constructs WTC and FLB.

		Speaking	Reading	Writing	Comprehension
Foreign language boredom	Pearson Correlation P-value N	−0.40^a^ <2.20 × 10^−16^ 649	−0.43^a^ <2.20 × 10^−16^ 649	−0.42^a^ <2.20 × 10^−16^ 649	−0.44^a^ <2.20 × 10^−16^ 649

a Correlation is significant at the 0.01 level (two-tailed).

As Table 6 demonstrates, all sub-constructs WTC are negatively correlated with foreign language boredom: speaking (r = −0.40，n = 649, p < 2.20 × 10^−16^, α = 0.01), reading (r = −0.43, n = 649, p < 2.20 × 10^−16^, α = 0.01), writing (r = −0.42, n = 649, p < 2.20 × 10^−16^, α = 0.01), and comprehension (r = −0.44, n = 649, p < 2.20 × 10^−16^, α = 0.01).

### 4.2 The second RQ

RQ2 How can the willingness to communicate of Chinese struggling EFL learners be anticipated by their foreign language anxiety and foreign language boredom?

**Fig. 1:**
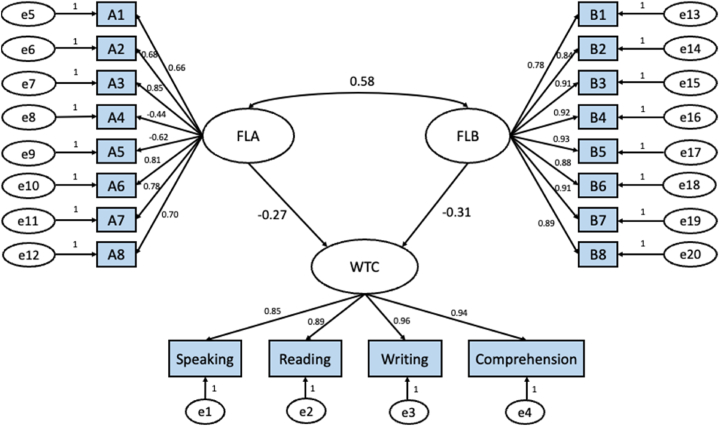
The model of the interplay among foreign language anxiety (FLA), foreign language boredom (FLB) and willingness to communicate (WTC).

In order to reply to the second research question, SEM was employed through package lavaan 0.6–9 on R 4.0.5. To check the merits of the causal interplays among the components, standardized evaluations were inspected. Fig. 1 indicates the model of the interactions among FLA, FLB and WTC. In it, A1, A2 … A8 stand for the items in *Foreign Language Classroom Anxiety Scale* (FLCAS), B1, B2⋯B8 represent the items in *Foreign Language Classroom Boredom Subscale* (FLCBS), and e1, e2 … e20 show the corresponding residuals.

As revealed in Fig. 1, both foreign language anxiety (β = −0.27, p < 2.20 × 10^−16^) and foreign language boredom (β = −0.31, p < 2.20 × 10^−16^) are negative significant predictors of their WTC, while foreign language anxiety correlates positively and significantly with foreign language boredom (β = 0.58, p < 2.20 × 10^−16^).

Finally, as can be seen from Table 7, goodness of fit indices was utilized to check the model fit. For a fit model, $\chi^{2}/df$ should not be more than 5, CFI, NFI and IFI are required to be above 0.90, RMSEA and SRMR need to be less than 0.08. Table 7 shows that all the fit indices are in a quite satisfactory level except that $\chi^{2}/df$ is slightly higher but acceptable.

**Table 7:** Goodness of fit indices.

	$\chi^{2}/df$	CFI	NFI	RMSEA	IFI	SRMR
Acceptable fit	<5	>0.90	>0.90	<0.08	>0.90	<0.08
Model	5.52	0.94	0.93	0.08	0.94	0.06

## 5 Discussion

The present study aimed to scrutinize the relation between foreign language anxiety (FLA), foreign language boredom (FLB) and willingness to communicate (WTC) in Chinese struggling EFL learners, and the predictive effects of FLA and FLB on WTC among these learners. Some important findings were discovered in the research.

Firstly, Chinese struggling EFL learners' FLA correlates positively and significantly with their FLB. This is consistent with the finding of Li et al. [19], which discovered that boredom is related to some negative feelings, including anxiety. This high correlation between FLA and FLB also echoes the results of the empirical study conducted by Li and Han [21] on some Chinese non-English major freshmen, whose foreign language anxiety and boredom were positively relevant. But as struggling EFL learners, the students majoring in music, physical education and fine arts in this current study had a higher correlation between foreign language anxiety and boredom (r = 0.52) than the non-English major freshmen (r = 0.379). This reveals the fact that, compared with other learners, Chinese struggling EFL learners' foreign language anxiety is more likely to trigger their foreign language boredom, or vice versa. This vicious cycle reflected in Chinese struggling EFL learners deserves special attention from language researchers.

Secondly, Chinese struggling EFL learners' FLA is negatively correlated with their WTC, and is a negative significant predictor of their WTC. This result lends support to previous study conducted by Price [66] who discovered that high-level anxious students did not have interest in communicating with others. It is also in line with the research carried out by Horwitz [11], which reported that foreign language anxiety rendered students to be unwilling to communicate in English. In the case of Chinese struggling EFL learners majoring in music, physical education and fine arts, their unique learning situation must be taken into consideration, that is, these students have a relatively low level of English proficiency. Many studies have confirmed that foreign language anxiety is notably associated with foreign language proficiency [67] and low-perceived foreign language proficiency is one of the factors leading to foreign language anxiety [37]. Understandably, Chinese struggling EFL learners' lower level of English proficiency leads to their higher foreign language anxiety, which further discourages them from being involved in communication related to the use of English.

Moreover, Chinese struggling EFL learners' FLB is negatively correlated with their WTC, and is a negative significant predictor of their WTC as well. This finding confirms the claim that feeling bored while studying was such a common phenomenon that a sign of boredom was the prevalent silence and disengagement in the classroom [68]. It also lends support to the study of Li et al. [19], which revealed the prevalent emotional experience of boredom among Chinese university students in learning English. However, the Chinese struggling EFL learners are even worse in this aspect. This is probably still due to these students' relatively low English proficiency since one of the causes of boredom is connected with limited ability to understand [53,69], which will increase the difficulty in understanding the communicative tasks, thus it is not surprising that these struggling students are unwilling to communicate.

It is noteworthy that although both FLA and FLB of these Chinese struggling students are negatively correlated with their WTC, their FLB and WTC have a higher negative correlation (r = −0.45) than their FLA and WTC do (r = −0.26). This is an important finding of this current study, which indicates that Chinese struggling EFL learners' foreign language boredom has a more deleterious effect on their WTC than their foreign language anxiety does. Language researchers and practitioners should pay due attention to this phenomenon and conduct further research to study it.

## 6 Conclusion

Taking the struggling EFL learners as the research objects, this present study conducted an empirical study on Chinese college students majoring in music, physical education and fine arts to examine the correlation between foreign language anxiety (FLA), foreign language boredom (FLB) and their willingness to communicate (WTC), and the predictive effects of these participants’ FLA and FLB on their WTC. It turned out that these students' FLA was highly and positively correlated with their FLB, while both their FLA and FLB were negatively linked to WTC and were negative significant predictors of their WTC. It deserves special attention that these struggling EFL learners felt more bored in studying English than those in other non-English majors, which had a deeper impact on their WTC.

All of these can be attributed to the relatively lower level of English proficiency of those struggling students. Extensive studies [6,37,53,69] have revealed that inadequate language proficiency, reflected in the poor academic achievement and performance, has a strong correlation with high foreign language anxiety and boredom, which leads to those students' unwillingness to communicate. Furthermore, the interplay among the three variables examined in the research — FLA, FLB, and willingness to communicate (WTC) — tends to establish a vicious cycle that significantly impedes the progress of those students' EFL learning. Then, how to lighten the struggling students' anxiety and boredom and enhance their willingness to communicate represents a formidable challenge for language researchers and practitioners alike.

Therefore, the findings in this present study may have significant pedagogical implications for future studies in the struggling EFL learners. The first suggestion is about the proper teaching material. In view of the actual language proficiency of those struggling EFL learners, relevant publishing houses should consider compiling special English learning textbooks suitable for them. And avid researchers may also conduct relevant studies in choosing appropriate teaching materials for those special EFL learners based on the investigation of their actual learning situation. Second, further studies may be conducted to explore the interpersonal relationship between teachers and those struggling students from various perspectives. Compared with other learners, struggling EFL learners need more confirmation, care and assistance from teachers to improve their psychological well-being and willingness to communicate. As a result, teachers need to be more caring, patient, enthusiastic and dedicated in dealing with those struggling EFL learners. Cao [70] found that a more positive and supportive relationship between teachers and students could lead to a higher WTC. Thus, many positive teacher behaviors including teacher care, teacher confirmation, teacher-student rapport, teachers' affective scaffolding as well as the practice of a loving pedagogy with the struggling EFL learners will be meaningful themes worthy of investigation [[71], [72], [73], [74], [75]]. In addition, educational fairness is significantly meaningful to those struggling learners. Since they have a lot more difficulty learning English in contrast with other EFL learners, they have a stronger desire to be treated fairly without any prejudice. Hence, teachers' respect and understanding will adequately induce their willingness to communicate in language learning. Finally, finding ways to organize communicative activities appropriate to those struggling students' proficiency level is a challenging task for teachers and researchers. It has been evidenced that boredom is related to activities which do not match the students’ proficiency level [51], and the topics under discussion would influence the extent of learners' WTC [58,59,76]. Therefore, designing activities in accordance with those struggling students' language proficiency and interest would be of practical value and will boost their enthusiasm in language learning as well, which will, in turn, facilitate their willingness to communicate, and ultimately lead to desirable academic achievement.

Nevertheless, it is essential to acknowledge certain limitations of this study. Firstly, while the empirical research employed in this study offers valuable insights, many other approaches may be utilized to fully analyze the factors affecting those struggling students' EFL learning. Derakhshan et al. [77], in their recent review, presented a range of emerging methods for analyzing and interpreting academic emotions, such as the process tracing approach, time series analysis, latent growth curve modeling, and Q methodology. Future studies focusing on struggling students could benefit from incorporating these innovative approaches. Secondly, the participants in the survey were undergraduate students from Chinese universities, thereby warranting further investigation involving struggling students from diverse educational levels and contexts. Since cultural value is an important factor affecting WTC [62,63], it is critical that more comparative studies be conducted by Asian researchers in countries like Japan, Thailand, or Korea with similar cultural backgrounds to further verify the upshots of the present study. Lastly, it is worth noting that the current study treated the students majoring in music, physical education and fine arts as a whole since they are generally deemed as struggling EFL learners in China in view of their unique learning experience. However, it is important to recognize that within these majors, there may be students who excel in English learning. Hence, such students may be studied separately in the future in terms of scaffolding theory [78], especially peer scaffolding [79] to investigate how proficient students can support their struggling classmates in making progress in EFL learning. Such investigations would be of significant importance in the realm of struggling EFL learner studies.

## Author contribution statement

Shuxia Bai: Conceived and designed the experiments; Performed the experiments; Analyzed and interpreted the data; Wrote the paper.

## Data availability statement

Data will be made available on request.

## Declaration of competing interest

The authors declare that they have no known competing financial interests or personal relationships that could have appeared to influence the work reported in this paper.
