# Supplementary material for: The predictive effects of foreign language anxiety and boredom on willingness to communicate among Chinese struggling EFL learners
Source: Heliyon. 2023 Aug 29;9(9):e19610. doi: 10.1016/j.heliyon.2023.e19610 (PMC10558861; doi:10.1016/j.heliyon.2023.e19610)
Supplement: Multimedia component 1 [file mmc1.docx]

**Chinese Struggling EFL Learners' Foreign Language Anxiety, Boredom and Willingness to Communicate**

**Participant Consent Form**

You are being invited to take part in a research study on Chinese struggling students' foreign language anxiety, boredom and willingness to communicate in the EFL (English as a foreign language) context. In this study, you will be asked to complete this questionnaire by reporting to what extent you agree with the following descriptions. It takes typically 5 to 8 minutes. You have the right to withdraw from the survey at any time without any negative consequences. There are no known risks for you and your participation is voluntary. We promise to keep all the collected data confidential only for research goals. If you have any questions about this research, you may contact the researcher (Shuxia Bai; Email address: baishx8968@henu.edu.cn). Please read the following declaration of participation and select "Yes" if you agree to participate in this questionnaire.

*I hereby declare that I voluntarily participated in this study. I let the researchers use my responses as data as far as my identity remains anonymous. In addition, the researchers guarantee that all information I provide for this study will be treated confidentially.*

**Consent**

○Yes

○No

**Demographic Information**

**1. Age**

____________________________

**2. School**

____________________________

**3. Gender**

○Male

○Female

**4. Level of Education**

○Freshman

○Sophomore

○Junior

○Senior

**5. Major**

○Music

○Physical education

○Fine arts

**Questionnaire:**

**Part 1:**

Instruction: The items of this scale measure your level of anxiety in foreign language classroom. All items are scored on a standard 5-point Likert scale ranging from 1 (strongly disagree) to 5 (strongly agree). Please indicate to what extent you agree with each statement.

**1. Even if I am well prepared for FL class, I feel anxious about it.**

**2. I always feel that the other students speak the FL better than I do.**

**3. I can feel my heart pounding when I’m going to be called on in FL class.**

**4. I don’t worry about making mistakes in FL class. (reverse-coded)**

**5. I feel confident when I speak in FL class. (reverse-coded)**

**6. I feel nervous and confused when I am speaking in my FL class.**

**7. I start to panic when I have to speak without preparation in FL class.**

**8. It embarrasses me to volunteer answers in my FL class.**

**Part 2:**

Instruction: The items of this scale measure your level of boredom in foreign language classroom. All items are scored on a standard 5-point Likert scale ranging from 1 (strongly disagree) to 5 (strongly agree). Please indicate to what extent you agree with each statement.

**1. The English class bores me.**

**2. I start yawning in English class because I’m so bored.**

**3. My mind begins to wander in the English class.**

**4. I am only physically in the classroom, while my mind is wandering outside the English class.**

**5. It is difficult for me to concentrate in the English class.**

**6. Time is dragging on in English class.**

**7. I get restless and can’t wait for the English class to end.**

**8. I always think about what else I might be doing to kill the time rather than sitting in this English class.**

**Part 3:**

Instruction: This scale is composed of statements concerning your willingness to communicate in English inside the classroom. All items are scored on a standard 5-point Likert scale ranging from 1 (almost never willing) to 5 (almost always willing). Please indicate to what extent you agree with each statement.

**Speaking in class, in English**

**1. Speaking in a group about your summer vacation.**

**2. Speaking to your teacher about your homework assignment.**

**3. A stranger enters the room you are in, how willing would you be to have a conversation if he talked to you first?**

**4. You are confused about a task you must complete, how willing are you to ask for instructions/clarification?**

**5. Talking to a friend while waiting in line.**

**6. How willing would you be to be an actor in a play?**

**7. Describe the rules of your favorite game.**

**8. Play a game in English, for example Monopoly.**

**Reading in class (to yourself, not out loud)**

**1. Read a novel.**

**2. Read an article in a paper.**

**3. Read letters from a pen pal written in native English.**

**4. Read personal letters or notes written to you in which the writer has deliberately used simple words and constructions.**

**5. Read an advertisement in the paper to find a good bicycle you can buy.**

**6. Read reviews for popular movies.**

**Writing in class, in English**

**1. Write an advertisement to sell an old bike.**

**2. Write down the instructions for your favorite hobby.**

**3. Write a report on your favorite animal and its habits.**

**4. Write a story.**

**5. Write a letter to a friend.**

**6. Write a newspaper article.**

**7. Write the answers to a "fun" quiz from a magazine.**

**8. Write down a list of things you must do tomorrow.**

**Comprehension in class**

**1. Listen to instructions and complete a task.**

**2. Bake a cake if instructions were in English.**

**3. Fill out an application form.**

**4. Take directions from an English speaker.**

**5. Understand an English movie.**
